# Care coordination for people living with serious mental illness: understanding the caregiver's perspective

**DOI:** 10.3389/frhs.2024.1473235

**Published:** 2025-01-07

**Authors:** Pamela Obegu, Kayla Nicholls, Mary Alberti

**Affiliations:** Canadian Institute for Advancements in Mental Health, Toronto, ON, Canada

**Keywords:** family caregivers, caregivers' support, serious mental illness, care coordination, system navigation, collaborative care

## Abstract

**Introduction:**

Family caregivers of people living with serious mental illness such as bipolar disorder, psychosis and schizophrenia, are continuously burdened with caregiving, following the complexities of navigating the mental health system for their loved ones. The aim of the study was to understand the perspectives of caregivers about care coordination for people living with serious mental illness, highlighting the current landscape and new directions across Canada.

**Methods:**

In this co-designed participatory qualitative research, caregivers of people living with serious mental illness, and service providers were engaged and purposively sampled across Canada.

**Results:**

The main findings of the study revealed care coordination as a key strategy to alleviate the burden of caregivers and enhance sustainable support for them. In complement with collaborative mental health care, care coordination can improve service delivery and strengthen the mental health system.

**Conclusion:**

Given the severity of bipolar disorder, psychosis and schizophrenia, it is important that we prioritize care for people living with these illnesses while providing support for their caregivers who bear the brunt of the otherwise fractured mental health system. Ultimately, collaboration between people and systems is how the mental health system can be much improved, and care coordinators serve as resourceful go-betweens in this ‘collaborativerse’.

## Background

Serious mental illnesses (SMIs) such as bipolar disorder, psychosis and schizophrenia, have many undesirable impacts not only on persons living with SMIs but also on their family caregivers (1, 2). Caregivers of individuals living with SMIs shoulder numerous responsibilities that transcend their conventional duties because the situation presents unique challenges that also transcend local boundaries (3). They navigate a complex landscape fraught with mental, economic, and logistical challenges while making concrete efforts to provide the best possible support to their loved ones in distress (3, 4). Globally, caregivers play key roles in supporting their family members with SMIs, providing care for them during their referral to mental health services, promoting the maintenance of regular contact with professional care, and supporting their adherence to prescribed medications (4, 5). The vital roles caregivers play in supporting their loved ones with SMI may sometimes be overshadowed by bottlenecks such as limited access to resources and support services, financial stressors, stigma, coordinating multiple appointments, and burnout from the demanding tasks associated with caregiving (6, 7). Additionally, the societal myths associated with SMIs such as stigma, can cause caregivers to isolate themselves socially, and by extension, not know where to find resources to care for their loved ones (7, 8). Caregivers of people living with SMIs frequently experience barriers in accessing the resources necessary to facilitate their caregiving roles effectively. Other previously reported barriers to mental health services in Canada include long waiting times, demographic and geographic inequities (e.g., people living in rural areas and Indigenous communities are typically isolated from locations where most mental health services are available), and stigma (9). Prolonged waiting time to see a psychiatrist particularly creates a disproportionate burden for the caregiver and their loved ones living with SMIs (8–10). For caregivers, the waiting time for access to psychiatric services often leaves them with the daunting task of managing the symptoms and behaviors of their loved ones devoid of professional support and guidance (8). For the person living with SMI, long waiting times can aggravate their symptoms, increase their risk of hospitalization, and delay their recovery (9). There remain a need to alleviate the suffering of caregivers of mentally ill persons as the expected support from health professionals has been perceived to be inadequate (11). In this situation, mental health care coordinators (also referred to as patient navigators, family navigators, system/service navigators) become indispensable.

Mental health care coordination is built on the constructs of the social prescribing model, wherein a dedicated capacity is made available to aid and empower individuals and populations to access adequate support. In addition to reducing readmissions, sustaining post-discharge care, managing a series of needs and social stressors that can impede an individual's mental well-being, thereby alleviating the pressures on the healthcare system (12, 13). In Canada, mental health care coordinators are often through not-for-profit organizations who work collaboratively with community resource persons, to facilitate caregivers/family members' access to relevant programs and services based on individual needs of their loved ones with SMIs (14). Also, existing mental health care coordinators in Canada are reportedly offering to provide services to parents of children living with SMIs through helplines, support groups and mobile one-on-one support (15, 16). With care coordination, caregivers are assisted with understanding their loved ones' concerning symptoms, finding resources, navigating services, and providing referrals and linkages (15, 16). A study on the experiences of caregivers as clients of patient navigators found that aside from caregivers' assumption of additional roles when caring for their dependents with mental health needs, they also experienced challenges and frustrations in a bid to navigate the various systems (17). These challenges include poor communication between care providers and structural barriers as caregivers continue to identify the need for help with navigating the system and knowledge acquisition (17, 18). By providing support and guidance, mental health care coordination initiatives aim to empower caregivers and build their capacity to navigate the challenging mental healthcare landscape. In navigating the mental health landscape, caregivers of people living with SMIs are assured of timely access to needed services, eradicating bureaucratic processes and long waiting times (12). Mental health care coordination also affords caregivers the benefits of enhanced coordination of care that ensures effortless continuity of care and support, thereby providing a considerable measure of support (12–14). For caregivers, working with mental health navigators “lifted the huge weight and took the emotional anxiety and stress away” (18). Some of these caregivers reported that their ability to care for their loved ones had improved due to the support and resources they received, which in turn improved their quality of life (18).

The concept of mental health care coordination is relatively new in Canada, with few coordination centers providing services to caregivers and their families. One of the earliest mental health care coordination concepts in Canada was a family navigation trial in Sunnybrook hospital in 2013, that helped youth struggling with mental health and/or addiction challenges (19). The trial proved successful with evidence of decreased waiting times, timely intervention, and by extension relief/support for caregivers, yet care coordination is still not widespread (19). In a rapid review summarizing the evidence of scale up/spread of coordination services, Waddel and colleagues (20) reported that “gathering evidence on the outcomes of coordination services being implemented” was part of the process in supporting the spread of coordination service (20). While the evidence has shown that caregivers feel supported when there is available and accessible mental health care coordination service (18), there is a lack of evidence examining mental health care coordination for people living with serious illness through the lends of the caregiver, who more than often has navigate care on behalf of the people living with SMIs. This study aims to explore the perspectives of caregivers on care coordination of people living with SMIs, highlighting the current landscape and new directions across Canada. This study is part of a larger study set to identify ways to enhance support for caregivers of people with SMIs across Canada.

## Methods

### Setting and participants

The study was carried out between July and October 2023, across provinces representative of Atlantic Canada, Central Canada, The Prairies, The Territories and The West Coast. Participants in the study were caregivers who were primarily providing care to cohabiting relatives/close friends living with schizophrenia, bipolar disorder or psychosis. In addition to caregivers as study participants, mental health service providers including registered clinical counsellors, social workers, and program directors of mental health organizations also participated in the study. This qualitative study employed a participatory research (PR) design approach, guided by interpretive description to participants' perspectives on how best to support family caregivers in their role as caregivers of people living with SMIs (21, 22). This approach allowed for participation and engagement of the study population throughout the study. The involvement of the study population in this first of a kind pan-Canadian caregiver study, promises a focus on support for the caregiver as opposed to the more explored focus on caregiving. We will explore the perspectives of caregivers as well as service providers on the topic of health care coordination for people living with SMIs across Canada, by learning practical lessons together.

### Recruitment and sampling

We leveraged our networks and contacted several caregiver organizations as well as service providers across provinces and territories in Canada, inviting them to an initial meeting. In attendance at this meeting were 40 caregivers and mental health service providers, representing 19 organizations, to whom we presented the proposed study. We deliberated the depth and breadth of the proposed study, its relevance, direction and expectations, and received feedback from attendees. Included in the received feedback was how best to address our proposed research objectives, including the suggestion to have caregivers and service providers together in focus group discussions to provide well-rounded perspectives on the topic, as this had never been done. We would learn at the meeting that service providers and caregivers were often on opposite ends of the divide and there would be value in discussing together as a group. We also received suggestions/contact information to contact more caregivers and service providers for the study and purposively sample study participants. This co-design component of the study was in line with the participatory research approach adopted by the study to enhance participation and co-learning for the benefit of the study population (23). Since the study was carried out in the summertime, we had several declined responses to participate in the study for reasons ranging from lack of substitute care among caregivers during study participation, to work/personal travel among service providers. Participation was on a voluntary basis and participants were made aware of their rights to decline at any time even during the interview. Participants signed a written informed consent form and agreed to be interviewed through videoconferencing. The study was carried out by the research team of the Canadian Institute for Advancements in Mental health (IAM) with funding from Petro-Canada's CareMakers Foundation. ADVARRA Institutional Review Board Services approved this study on the 13th of July 2023 (Pro00072543).

### Data collection and analysis

Data collection involved one focus group discussion with both caregivers and service providers (ten participants and two group facilitators); and fifteen key informant interviews with only caregivers. Inclusion criteria for the focus group participants were as follows: the caregiver must be 18 years of age and be a family caregiver (i.e., a parent, spouse or other relative) or a close friend of a person living with a serious mental illness; OR, an adult currently working as a mental health service provider (including registered clinical counsellors, social workers, and program directors of mental health organizations), in either a public or not-for-profit organization. Both focus group discussion and key informant interviews were audio-recorded. The focus group discussion lasted 90 min, while the key informant interviews lasted 45–60 min. All audio recordings were transcribed verbatim, and the anonymity of participants was maintained through the removal of identifying information of participants. The de-identified data was validated by participants. All datasets were reviewed and coded and maintained a codebook with code names and definitions. Data was analyzed using NVivo software version 12 (24). After extensive review of the codes, similar codes were formulated into four main themes according to the tenets of qualitative research (25), with the aim of effectively identifying and summarizing the experiences of participants. We relied on the multiple perspectives of the research team to establish necessary rigor, credibility and detail for understanding the study findings.

## Patient and public involvement

Caregivers were involved in this study through research design and interpretation of data.

## Results

The study involved a total of 25 participants (*n* = 18 caregivers, *n* = 7 service providers). Among the caregivers were mostly female (*n* = 14). The longest time spent caring was 31 years, and the shortest time was 4 years. We identified four themes that described the current landscape of care coordination including participants' experience around mental health care coordination: (1) “I had to find everything on my own”; (2) “We need a road map”; (3) *The only way out of this is a collaboration between people and systems*; (4) “Nobody follows up with this stuff”.

### “I had to find everything on my own”

Participants echoed in agreement on how difficult accessing mental health services typically is for their family members/loved ones living with SMIs. A caregiver said, “*I had to find everything on my own*” (FG3) and a service provider underlined that, “*I think, and other people have brought this up, that mental health is not covered under the Medicare system in Canada, and so that's such a barrier to finding help or anything at all. And even though we are a not-for-profit organization, the fees that we need to charge to keep our doors open are beyond what a lot of folks can afford*” (FG8). Another caregiver described how they began their caregiving journey not knowing where to look:

“I was first in shock learning that my child has this very severe illness, what to do, where to go. Not giving me a few pamphlets and telling e to go home and read and learn. That's so not okay—it's such a complicated system. Because growing up, we knew we can go to the doctor, we can go to the hospital when we’re sick, but with this illness there's no such thing and people can’t get their head wrapped around this, you know, until you’re in it and in the chaos. So now you’re trying to navigate a system when your son or daughter is in a psychotic state and your household is probably in chaos and your mind is in chaos, but all you have is a pamphlet that you got to learn with no direct access to resources for help. So, you have to figure out on your own how to navigate a whole new system that you had no idea how to navigate. It is hard”. (K4)

Many participants pointed to the justice system as their first encounter with finding help for their loved ones. A caregiver noted that “*many parents of children with severe mental illness get their first help after our children got involved with the justice system because with psychotic breaks, they almost always get caught up in the weeds of the law*”. (K10)

“My son was thrown in jail for an otherwise small offense, and they said something was seriously wrong and he managed to see a psychiatrist while in prison. That was my first inclination of just what I was up against. The psychiatrist told me that he suspected schizophrenia. So anyway, I ended up in a court hearing. I was able to turn the judge around. I asked him to help me help my son, so we got the psychiatric assessment that we’d been looking for. He ended up in [name of mental health institution withheld] and I was in the hospital a lot for the next four months. He was diagnosed and the diagnosis was severe schizophrenia, many negative symptoms, behavioural complications, etc.”. (K9)

A caregiver also noted how beneficial peer support groups were with identifying and accessing available services: “*I learned from peer support where to find what in the early days of my daughter's illness. They (peer support group) provided educational resources, and I eventually learned that caregiving is a lifetime role*” (K11). Another caregiver added that, “*peer support was quite something. I was looking for help, someone to help me navigate the system, but peer support was a good place to start*” (FG2).

### “We need a roadmap”

Caregivers across provinces emphasized the need for direction with the provision and delivery of mental health services. A caregiver described this need saying, *“family members need a roadmap. We need something. We need a roadmap when we are told that my child has schizophrenia”. How do I do this. Where do I go, where do I get help, where do I get resources because I don’t know. And it's only if you come across someone that says, “Oh, why don’t you go here”. “Oh, why don’t you call this number”, you don't know where to go because everywhere that you go, you’re told, “Oh, there's a waiting list. Oh, they don’t meet the criteria because they have schizophrenia”.* (K12)

“Dealing with the hospital system should be less complicated than it is. Referrals should be much smoother than they are”. (K6)

Participants noted that when one typically presents at the emergency department, they are guaranteed care irrespective of the waiting time, but this was not the case with their loved ones living with SMIs. A caregiver shared that, “*severe mental illness is not like any other illness. When we are in the emergency unit to be seen by somebody, by the time we are seen, there will be no bed left in psychiatry for us and we get turned back. An emergency unit to just deal with our clients, for our people that have serious mental health illness, away from the huge congregation room of people waiting for services, is a good place to start*” (FG4). In agreement, a service provider added that, “*it does not help our clients to have to sit with so many other people that are crying and upset and throwing up and all, and clients get agitated. They don't like to sit with that many people, because it generally riles them up and so, even if they had a separate area in the emergency (department), when they are in crises, that is a fair ask*” (FG10).

In addition to needing care coordination, and a section of the emergency department dedicated to people living with SMIs, participants all echoed their need to be involved in the hospitalization and discharge process. Involvement ranged from being communicated with respectfully by physicians who should regard them as “*the foundation of knowledge of what is going on with their loved ones living with SMI*” (K6) to being handed a discharge plan.

“It is important for caregivers to be part of the discharge plan”. (K11)

“No advice, no information after discharge. Nothing”. (K4)

“I was once told by a psychiatrist that the purpose of the hospital is to stabilize him (son) and kick him out. So, everything then falls on me”. (K7)

“I think value for the caregiver is to receive respect from the medical society. From the doctors, from the professionals because I don’t think caregivers feel that because of the way they get spoken to, or they don’t get spoken to, or they get dismissed. And specifically, when you take your loved one to an emergency department you get dismissed because all they want to do is take them in, give them some medication, make them stable and get them out”. (K12)

### “The only way out of this is a collaboration between people and systems”

Participants described their experience with mental health care coordinators and how it has provided them support or the lack thereof. A participant from Manitoba stated that, “*the expectation here is for caregivers to navigate the system through navigators which is a paid position. We have care coordinators here in Manitoba that do that job, but we need more of them*” (Participant from MB). Two participants from British Columbia noted that their cities hired “care coordinators” who have helped with care coordination from a social determinants of health perspective, for people living with SMIs and their caregivers.

“The City hired a person to address homelessness in the community, which is very progressive, and they coordinate services for people” (Participant from BC).

“Very progressive but there needs to be more of this” (Participant from BC).

A participant from Saskatchewan noted the effect of insufficient community resources saying, “*we generally have community services. We have some that are very, very good, but we don't have enough. And it's hard to have a community worker that can help them (caregivers and their loved ones living with SMIs) often enough, and the community services are overwhelmed as well. So, certainly community resources need to be in place as well”* (Participant from SK).

From Quebec, participants described the navigation support they received from a known caregiver group, describing the caregiver organization as “*an advocate for caregivers who point caregivers to care coordination within the province despite insufficient funds to keep offering support to caregivers*”. (Participant from QC).

Participants from Newfoundland and Prince Edward Island expressed the lack of care coordination in their provinces which reflected the renowned shortage of doctors and nurse practitioners despite being small provinces.

“As a service provider, I find there are a lot of barriers to being able to support caregivers with care coordination. We are a small province but have a shortage of doctors and nurse practitioners” (Participant from PEI).

“If there's a network trying to be built around the person (living with SMI), the barrier is often, who is taking charge of this, who's taking the lead on this, who do we send them to, who best suits this to thread it through. And that's a big, big challenge and it's a big barrier here, there's no starting point” (Participant from NFL).

From Ontario, there were echoes of skeletal care coordination through independent organizations who grappled with shortage of psychiatrists and consequently, long lists of caregivers needing care coordination.

“The only way out of this is a collaboration between people and systems. There was a navigation trial in Sunnybrook Hospital years ago that worked. We need to replicate it across the province” (Participant from ON).

“There is need for trained mental health workers tailored to caregivers who know what we need” (Participant from ON).

“Even with the service phone lines, there is the problem of finding someone to talk to and there is the foreseeable time when they get up and leave” (Participant from ON).

### “Nobody follows up with this stuff”

As participants expressed their frustrations with finding help with care coordination, most of them pointed to the lack of organized planning and monitoring of funding as a barrier to adequate mental health service delivery and ultimately care coordination.

“You know, they (government and policymakers) talk a lot about putting money into mental health but where is it going, who knows”. (K12)

“You know what another problem is, I talked to the president of the [name of mental health not-for-profit organization], we were at the same charity event maybe eight or ten years ago. And she said the problem is the funding comes in and there's no organization of where this funding goes so you have a lot of overlapping programs all over the place. So, a little bit here, a little bit there, and nothing substantial. Nobody follows up with this stuff, it's a lot of duplication and there's so many parts of this that need to be fixed from the funding, to where it's going, to the planning and monitoring”. (FG10)

In the same vein, a caregiver described how organized funding being directed to training of mental health care coordinators/service providers will be a great support for caregivers.

“I feel that the government needs to put more money into mental health services and more mental health workers and stop relying on police because, the police aren’t trained to handle serious mental health issues even though they claim they are. Like we’ve had run-ins with the police and it's not very pleasant, that's for sure. So, I just feel like the money should go to the training of more people helping out in the mental health field, which would definitely be helpful for me as a caregiver, because then I would know that if we turn somewhere, we will get help”. (K4)

## Discussion

The present study describes the perspectives of caregivers as well as service providers about care coordination for people living with SMIs in Canada. Our findings show that caregivers of people living with SMIs start out with no information, no readily available services and support when they are first made aware of their loved ones' serious mental illness. When medical attention for people living with SMIs is sought, which is often through getting caught up with the law and justice system as this present study found, the lack of appropriate and adequate care leaves a huge service delivery gap for caregivers who often bear the brunt of this identified system failure. Both focus group participants and key informant interviewees expressed an urgent need for mental health care coordination. This expressed need was a proposed strategy to address the prolonged period of time it takes to see a psychiatrist as typically experienced by caregivers and their loved ones living with SMIs (8, 10). The identified link between prolonged waiting time to see a psychiatrist and shortage of doctors across Canada can further be addressed through collaborative care “shared model”, where mental health professionals will determine among themselves whom and how to best provide specific aspects of care to patients (26). For the person living with SMI, long waiting times can aggravate their symptoms, increase their risk of hospitalization, and delay their recovery. This in turn burdens the caregiver who has to help manage their loved ones while waiting for help whenever it comes, if it comes. Given the severity of schizophrenia, psychosis and bipolar disorder, the lack of a systemic approach to care is alarming for people living with SMIs and their family members. While provincial governments are responsible for health care in Canada, our findings reveal the fractured nature of health care for people living with serious mental illness as a common challenge across Canadian provinces. Mental health care coordination was proposed as a strategy to help caregivers know where and how to start their caregiving journey in addition to alternative yet timely resources to help during long waiting times. Caregivers always end up having to navigate the system for help for their loved ones, therefore, care coordination is the ideal kind of support for caregivers as they noted in the study, adding that it will alleviate their caregiving burden. This was similar to a finding of Luke and colleagues (17) in a study that captured the experiences of caregivers and their take on “patient navigation” (17). When study participants were engaged across Canadian provinces on their experience with mental health care coordination, the study revealed that most of the care coordination received were from non-governmental agencies with exception of participants from British Columbia. Participants from British Columbia identified some municipal governments' initiatives of hiring navigators, who took on care coordination from a social determinants of health perspective such as addressing housing issues for people living with SMIs. This revealed a need for government health departments to be more involved in mental health care coordination as well as scale up of care coordination. To this point, participants expressed a need to train more mental workers to be able to provide care coordination. Our findings also reveal that a caregiver organization in Quebec as described by participants, advocates for caregivers and points caregivers to care coordination across the province. This appears to be a great avenue for provincial governments to collaborate with known caregiver groups across provinces to scale up care coordination for caregivers of people living with SMIs. A common challenge across provinces was the shortage of doctors and psychiatrists as our study revealed in addition to several other studies (9, 10). While mental health care coordination alone cannot replace the shortage of psychiatrists and doctors across the country, our findings suggest that making available mental health care coordination and the scale up of it can help soften the effects of this specialized labour shortage through access to other resources, especially when delivered through a social determinants of health perspective as seen in British Columbia.

Another insightful finding of this study was the organization, planning and monitoring of funds for mental health services. Service providers and caregivers alike identified the need for a more streamlined funding pathways for mental health services to not only minimize overlapping mental health programs that exist, but enhance impactful service delivery. Again, earmarking funds for the training of workers to help with care coordination for caregivers and their loved ones living with SMIs was identified as a key support for caregivers across provinces. This arose in opposition to the involvement of law enforcement as is the case with people with SMIs and their caregivers early on in their caregiving journey. The involvement of law enforcement was not described by participants in positive light, which is in tandem with evidence showing that law enforcement officers are typically not well-equipped to intervene in situations involving people with SMIs and their caregivers which is consistent with a study by Chen et al. (27). The study found that police officers often thought of people living with SMIs as “difficult to manage and feared them more than those of the general public”. Participants expressed a need for a road map to mental health service delivery especially for people living with SMIs in light of the severity of serious mental illness. At the core of the road map is mental health care coordination to help with finding specialists to curb the dire effects of prolonged waiting times on severe mental illnesses, resources for coping with as well as the ongoing management of their loved ones. Participants described the need for a discharge plan beyond just being “kicked out of the hospital” or otherwise discharged. The place of mental health care coordination is much needed in the transition between hospital discharge and integration in the community for both caregivers and their loved ones. This is similar to a finding by Ojo and colleagues (28), in a study that identified aspects of mental health care coordination as effective strategies in ensuring continuity of care and supporting sustained recovery for discharged psychiatric patients (28).

## Conclusion

This study found mental health care coordination as an essential strategy in supporting caregivers and their loved ones living with serious mental illness. Mental health care coordination has shown to be a great tool, serving as a roadmap for caregivers to begin their caregiving journey and continued support beyond hospital discharge of their loved ones. The study identified reported progress in Canadian provinces where governments were more involved in care coordination through hiring care coordinators/navigators to help caregivers with resources to best support their loved ones living with SMIs. While there is a shortage of health providers in Canada, care coordination can help point caregivers to alternative resources that can curb long waiting times for people with SMIs, and possibly ameliorate their SMI conditions. Investing in the training of care coordinators can improve mental service delivery, and efficient management of funds for mental health service provision. Ultimately, “collaboration between people and systems” is how mental health systems can be strengthened, and care coordinators serve as the resourceful go-between in this ‘collaborativerse’.

### Strengths and limitations

In the present qualitative study, we employed a co-design participatory design, with members of the study population (caregivers who are often service users) directly working together with service providers to inform, guide and produce more meaningful research questions and methods. Another strength of the study is our nationwide study participation, covering both service users' and service providers' experiences with mental health care coordination in all regions in Canada. This elicited unique and rich findings around the research topic that to our knowledge had not been explored. However, the sample size is small, which indicates a low response rate thereby limiting generalizability. Since the study was carried out in the summertime, we had several declined responses to participate in the study for reasons ranging from lack of substitute care among caregivers during study participation, to work/personal travel among service providers.

## Data Availability

The qualitative data generated and analyzed in the current study are not publicly available due to patient privacy and legal restrictions. Requests to access the datasets should be directed to knicholls@iamentalhealth.ca.
